# Marktstrukturen und Wettbewerb im Kontext wirtschaftlicher Transformation

**DOI:** 10.1007/s10273-022-3312-3

**Published:** 2022-11-30

**Authors:** David Benček, Hannah Rosenbaum, John P. Weche

**Affiliations:** Monopolkommission, Kurt-Schumacher-Str. 8, 53113 Bonn, Deutschland

**Keywords:** L1, L4, L5, D4

## Abstract

Die deutsche Wirtschaft steht vor großen Herausforderungen. Neben Digitalisierung und Klimawandel wird im Zuge der COVID-19-Pandemie und des russischen Angriffs auf die Ukraine auch eine mögliche Deglobalisierung diskutiert. Diese Entwicklungen wirken sich nicht zuletzt auf bestehende Marktstrukturen und den Wettbewerb aus. Eine gesamtwirtschaftlich abnehmende Wettbewerbsintensität kann weitreichende makroökonomische Folgen haben, die bereits seit längerem diskutiert werden. Gegenstand der Diskussion sind etwa abnehmende private Investitionen, eine sinkende Lohnquote, eine steigende Einkommensungleichheit, ein abnehmendes Produktivitätswachstum sowie Auswirkungen auf die Inflation.

## Abstract (en)

The German economy is facing major challenges. In addition to digitization and climate change, a possible deglobalization is also being discussed in the wake of the COVID 19 pandemic and Russia’s attack on Ukraine. In particular, the disruptions and uncertainties recently observed in international value chains provide an incentive for companies to rethink and restructure supply chains. The re-nationalization of production processes contributes to greater security of supply, but sometimes at the price of higher production costs. Ultimately, these developments can affect existing market structures and competition. A decrease in the intensity of competition in the economy as a whole could have far-reaching macroeconomic consequences. Among those are, for example, declining private investment, a declining wage share, increasing income inequality, declining productivity growth and effects on inflation.

Aus wettbewerbsökonomischer Perspektive besteht nicht zwangsläufig ein Zusammenhang zwischen der Konzentration der Marktanteile aller Anbieter auf einem Markt und dem bestehenden Wettbewerbsdruck. Zur Beurteilung der Wettbewerbsintensität sind daher weitere Indikatoren notwendig. Nichtsdestotrotz stellen Marktkonzentration und Unternehmensgröße eine wichtige und wettbewerbsrelevante Kennzahl dar, die zudem im Rahmen der Wettbewerbspolitik aktuell wieder zunehmend Beachtung findet.[1]

## Gesamtwirtschaftliche Unternehmenskonzentration

Eine traditionell oft verwendete Kennzahl zur Einschätzung der Wettbewerbsintensität ist die Anbieterkonzentration. Ein deutlicher Anstieg der Unternehmenskonzentration in den USA hat die Diskussion um einen gesamtwirtschaftlich abnehmenden Wettbewerb und die möglichen Ursachen sowohl in den USA als auch im europäischen Binnenmarkt zuletzt befeuert (Grullon et al., 2019; EU-Kommission, 2020b). Die Monopolkommission hat seit 1974 den gesetzlichen Auftrag, Stand und Entwicklung der Unternehmenskonzentration und des Wettbewerbs in Deutschland zu beurteilen. Im aktuellen Hauptgutachten kommt sie zu dem Ergebnis, dass sich die Entwicklung der Unternehmenskonzentration und der Preisaufschläge – der Differenz zwischen dem Preis eines Gutes und den Grenzkosten seiner Produktion – in Deutschland insgesamt moderat darstellt.[2] Abbildung 1a zeigt die Entwicklung der Unternehmenskonzentration für die Gesamtwirtschaft und für einzelne Sektoren in Deutschland anhand von Trend und Verteilung des Herfindahl-Hirschman-Indexes (HHI) zwischen 2007 und 2019.[3] Für die Gesamtwirtschaft zeigt sich zwischen 2007 und 2013 ein leichter Anstieg der Un-

1 So wird in Deutschland z.B. im Rahmen der anstehenden 11. Novelle des Gesetzes gegen Wettbewerbsbeschränkungen (GWB) eine missbrauchsunabhängige Entflechtung diskutiert (BMWK, 2022). Auf europäischer Ebene ist in diesem Zusammenhang die Diskussion um das „New Competition Tool“ zu nennen (EU-Kommission, 2020a).

2 Für eine ausführliche Erläuterung der Vor- und Nachteile dieser Kennzahlen, Monopolkommission 2022, Abschnitte 2.1.1 und 2.2.1 in Kapitel I.

3 In die Berechnung des HHI fließen die Umsatzanteile aller Unternehmen eines 4-Steller-Wirtschaftszweiges ein. Im Extremfall mit nur einem Anbieter liegt der HHI bei 10.000 Punkten. Der Wert wird umso kleiner, je mehr Anbieter aktiv sind. In der EU dient ein HHI-Wert von 2.000 entsprechend den Regularien der Fusionskontrolle als Schwelle, bis zu der Märkte tendenziell noch als moderat konzentriert eingestuft werden. Der HHI wird von der Monopolkommission auf Basis der Wirtschaftszweigklassifikation der amtlichen Statistik (WZ 2008) berechnet.

**Dr. David Benček** ist Senior Analyst im wissenschaftlichen Stab der Monopolkommission.

**Hannah Rosenbaum** war Praktikantin bei der Monopolkommission und studiert Volkswirtschaftslehre an der Universität Mannheim.

**Prof. Dr. John P. Weche** ist Professor für Volkswirtschaftslehre und Standortpolitik an der Hochschule für Angewandte Wissenschaften Hamburg und Senior Analyst im wissenschaftlichen Stab der Monopolkommission.

Abbildung 1

**Trends und Höhe von Unternehmenskonzentration und Preisaufschlägen in Deutschland**

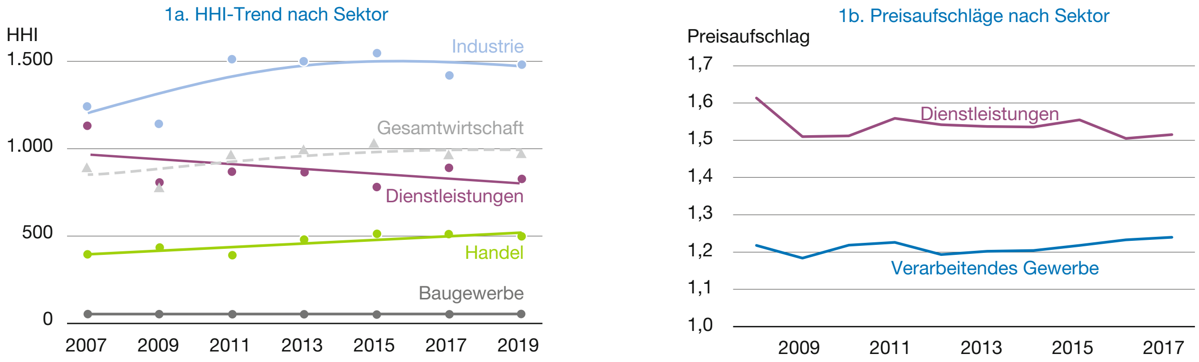

Sektorale Trends und jährliche Mittelwerte in Teilabbildung a) basieren auf HHI für 4-Steller-Wirtschaftszweige nach WZ 2008 und sind umsatzgewichtet. Trends entsprechen REML-Schätzungen von GAM-Modellen mit kubischen Splines. Industrie in a) bezeichnet Wirtschaftszweige in den Abschnitten B, C, D und E, verarbeitendes Gewerbe in b) nur Abschnitt C gemäß WZ 2008; Dienstleistungen setzen sich zusammen aus den Abschnitten H, J, L, M, N und Abteilung S95, Handel aus Abschnitt G und das Baugewerbe aus Abschnitt F.

Quelle: eigene Darstellung nach Monopolkommission (2022), basierend auf einer konzentrationsstatistischen Sonderaufbereitung des Statistischen Bundesamtes auf Basis des Unternehmensregisters sowie der AFiD Panel Industrieunternehmen 2008–2017 und Strukturerhebung im Dienstleistungsbereich 2008–2017.

ternehmenskonzentration, während zuletzt tendenziell eine Stagnation auf moderatem Niveau zu beobachten ist. In den einzelnen Sektoren können die Entwicklungen unterschiedlich sein. Auf dem höchsten Niveau liegt die Unternehmenskonzentration im Industriesektor, für den ähnlich wie für die Gesamtwirtschaft zunächst ein positiver Trend und schließlich eine Stagnation zu erkennen sind. Die anderen betrachteten Sektoren – Dienstleistungen, Handel und Baugewerbe – weisen sowohl im Vergleich zum Industriesektor als auch zur Gesamtwirtschaft eine geringere Unternehmenskonzentration auf. Hier zeigt nur der Handel einen positiven Trend in der Konzentrationsentwicklung – der HHI ist zwischen 2007 und 2019 um rund 30 % angestiegen. Für den Dienstleistungssektor wird zwischen 2007 und 2019 ein Rückgang der Unternehmenskonzentration um 17 % beobachtet und für das Baugewerbe eine durchgehende Stagnation auf einem niedrigen Niveau nahe Null. Eine hohe Marktkonzentration kann zwar ein Hinweis auf verringerte Wettbewerbsintensität sein, genügt für sich genommen aber noch nicht für eine entsprechende Schlussfolgerung.[4]

## Entwicklung der Preisaufschläge

Zusätzlich zur Unternehmenskonzentration wirft die Monopolkommission auch einen Blick auf die Entwicklung der Preisaufschläge von Unternehmen in Deutschland (vgl. Abbildung 1b). Preisaufschläge sind als Wettbewerbsindikator der Konzentration grundsätzlich vorzuziehen, da sie einerseits nicht von einer adäquaten Marktabgrenzung abhängig sind und andererseits direkt an der Marktergebnisseite ansetzen. Es zeigt sich ebenfalls eine ausgeprägte Heterogenität zwischen den Sektoren. Während die Preisaufschläge im Dienstleistungssektor zwischen 2008 und 2017 um rund 6 % gesunken sind, weisen die Preisaufschläge im verarbeitenden Gewerbe im selben Zeitraum einen Anstieg von rund 1,8 % auf. Unabhängig von den Sektoren verzeichnen vor allem Großunternehmen und Unternehmen in stark konzentrierten Branchen, z. B. in der Kokerei und Mineralölverarbeitung, wachsende Preisaufschläge (Monopolkommission, 2022). Ob diese hohen Preisaufschläge auf Marktmacht oder auf einen höheren Investitionsbedarf hinweisen, lässt sich aus diesen Daten allein nicht ableiten.

## Investitionsbedarf in immaterielles Vermögen

Hinsichtlich der Auswirkungen der Digitalisierung auf den Wettbewerb zeigen empirische Untersuchungen bisher keine eindeutige, sektorübergreifende Beziehung, sondern legen einen marktspezifischen Zusammenhang nahe (z. B. Bajgar et al., 2019; Calligaris et al., 2018, 2021; Weche und Wagner, 2021; Ferschli et al., 2021).[5] Ein bedeutender Treiber von Preisaufschlägen im Zuge der Digitalisierung kann die zunehmende Relevanz von immateriellen Vermögenswerten wie Patenten, Marken, Lizenzen sowie unternehmensspezifischem Humankapital sein. Wenn Unternehmen mehr in immaterielle Vermögenswerte investieren, um ihre Produk-

4 Für eine ausführliche Erläuterung des Zusammenhangs zwischen Unternehmenskonzentration und Wettbewerb siehe Heidorn und Weche (2021).

5 Die Digitalisierung von Unternehmen kann grundsätzlich entgegengesetzte Effekte auf die Wettbewerbsintensität haben. Für eine ausführliche Erläuterung vgl. Monopolkommission (2022).

Abbildung 2
**Investitionen in immaterielles Vermögen**

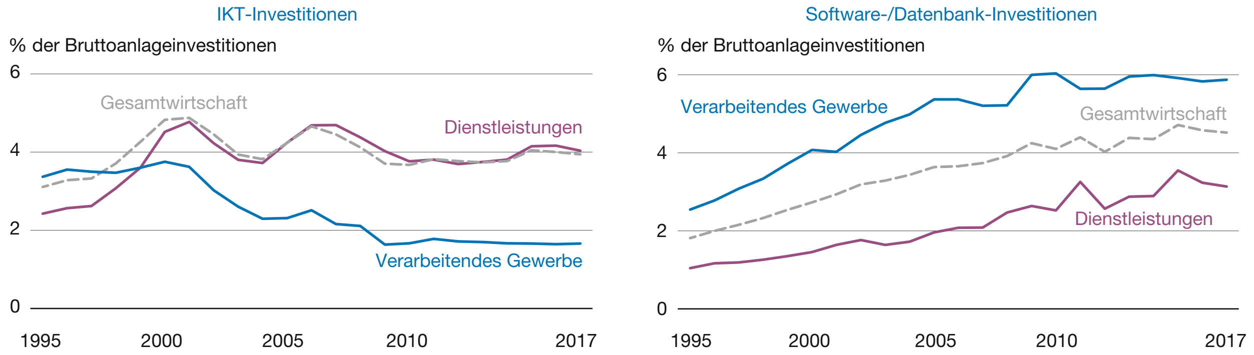

Quelle: KLEMS, eigene Berechnungen.

tivität zu steigern und einen technologischen Vorsprung gegenüber Konkurrenten zu erlangen, steigen ihre Fixkosten für die Produktion an. Dies kann dazu führen, dass Unternehmen höhere Preisaufschläge generieren müssen, um die gestiegenen Kosten decken zu können. Somit können auch hohe Fixkosten zu hohen Preisaufschlägen führen, womit letztere nicht zwangsläufig auf einen Anstieg von Marktmacht hindeuten (Berry et al., 2019; Van Reenen, 2018).[6]

Abbildung 2 zeigt, wie sich in diesem Zusammenhang die Investitionen in immaterielle Vermögenswerte zwischen 1995 und 2017 in Deutschland entwickelt haben. Konkret abgebildet sind die Investitionen in Informations- und Kommunikationstechnologie (IKT) sowie Software und Datenbanken als Anteil an den gesamtwirtschaftlichen Bruttoanlageinvestitionen sowohl für die Gesamtwirtschaft als auch aufgeteilt nach Dienstleistungssektor und verarbeitendem Gewerbe. Die Entwicklung der IKT-Investitionen zeigt klare Unterschiede gegenüber den Investitionen in Software und Datenbanken. Während die IKT-Investitionen lediglich Ende der 1990er und Anfang der 2000er merklich gestiegen und im verarbeitenden Gewerbe seither sogar deutlich gesunken sind, präsentiert sich für Software- und Datenbankinvestitionen ein kontinuierlicher Anstieg, sowohl für die Gesamtwirtschaft als auch in beiden Sektoren. Hierin spiegelt sich die Digitalisierung und die dabei wachsende Bedeutung immateriellen Vermögens bei deutschen Unternehmen wider. Insgesamt liegt Deutschland bei Digitalisierungsinvestitionen allerdings EU-weit lediglich im Mittelfeld, wie Abbildung 3 hervorhebt.

## Rolle der Digitalisierung

Zur Einschätzung der Rolle der Digitalisierung auf den Wettbewerb kann der Zusammenhang zwischen Digitalisierung und Preisaufschlägen betrachtet werden. Die Monopolkommission (2022) kommt in einer Regressionsanalyse auf Basis amtlicher Unternehmensdaten zu dem Ergebnis, dass die Preisaufschläge in digitalisierten Wirtschaftszweigen des verarbeitenden Gewerbes in Deutschland im Durchschnitt rund 3 % höher ausfallen.[7] Dieser Zusammenhang stellt sich in digitalisierten Wirtschaftszweigen des Dienstleistungssektors negativ dar: Unternehmen in überdurchschnittlich digitalisierten Dienstleistungsbranchen weisen 7,5 % niedrigere Preisaufschläge auf. Unter weiterer Berücksichtigung der Produktivität bleibt der geschätzte Zusammenhang im verarbeitenden Gewerbe nahezu unverändert, der Unterschied zwischen digitalintensiven und weniger digitalintensiven Branchen im Dienstleistungssektor vergrößert sich nun jedoch. Diese Ergebnisse könnten darauf hinweisen, dass Digitalisierung im Dienstleistungssektor durchschnittlich wettbewerbsfördernde Effekte entfaltet, während sie im verarbeitenden Gewerbe durchschnittlich zur Zunahme von Marktmacht führt.

In einer ähnlichen Analyse wird beobachtet, dass steigende IKT-Investitionen mit höheren Preisaufschlägen im Dienstleistungssektor und niedrigeren Preisaufschlägen im verarbeitenden Gewerbe einhergehen, während sich für steigende Softwareinvestitionen das umgekehrte Bild ergibt.[8] Dies könnte darauf zurückzuführen sein, dass sich der Dienstleistungssektor und das verarbeitende Gewerbe in ihren Strukturen grundsätzlich voneinander unterscheiden, könnte aber auch auf unterschiedliche Auswirkungen von immateriellen und materiellen Investitionen hindeuten.

6 Für eine ausführlichere Analyse der potenziellen Zusammenhänge vgl. Monopolkommission (2022).

7 Unter den Begriff „digitalisierte Wirtschaftszweige“ fallen hierbei alle Wirtschaftszweige, deren Digitalisierungsgrad, gemäß einer Klassifikation der OECD für 2-Steller-Wirtschaftszweige, über dem Median liegt (Flavio et al., 2018).

8 Es handelt sich um Ergebnisse aus Schätzungen von Regressionsmodellen mit Daten zur Digitalisierung von 2-Steller-Wirstchaftszweigen der KLEMS-Datenbank (Monopolkommission, 2022). Es wird geschaut, welche Veränderungen in den Preisaufschlägen mit um 1 % höheren IKT- bzw. Softwareinvestitionen einhergehen. Die Korrelationen sind in der Größenordnung von einstelligen Prozentzahlen.

Abbildung 3

**Deutschland bei privaten Digitalisierungsinvestitionen EU-weit im Mittelfeld**

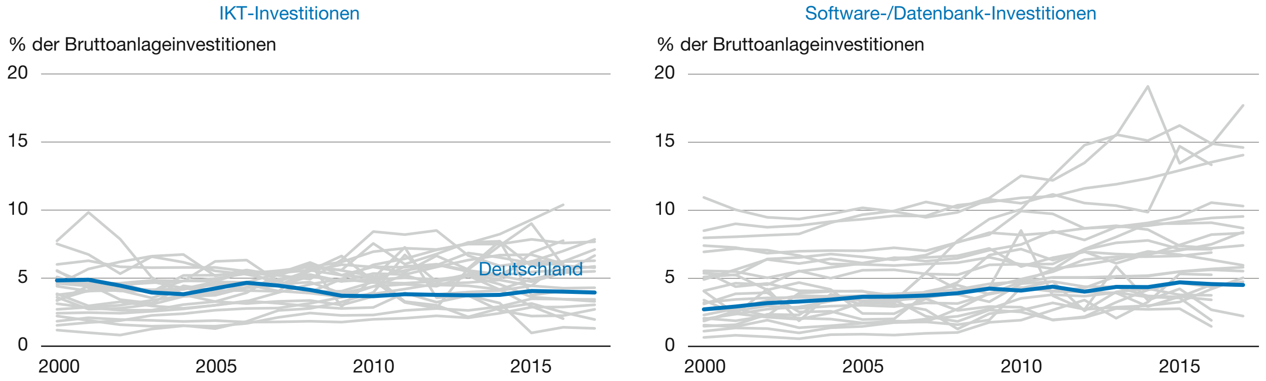

Gesamtwirtschaftliche Investitionen pro Jahr und EU-Mitgliedsland. Graue Zeitreihen stellen die Entwicklungen in anderen EU-Staaten dar. Daten zu Software-/Datenbank-Investitionen sind für 25 Staaten verfügbar, zu IKT-Investitionen für 23 Staaten.

Quelle: KLEMS; eigene Berechnungen.

## Berücksichtigung ökologischer Nachhaltigkeitsziele

Ein weiterer wichtiger Transformationstreiber neben dem technologischen Fortschritt ist der Klimawandel. Die Monopolkommission (2022) sieht keinen grundsätzlichen Zielkonflikt zwischen Wettbewerbsschutz und dem Erreichen von ökologischen Nachhaltigkeitszielen. Vielmehr ermöglicht ein funktionierender Wettbewerb die Durchsetzung innovativer und nachhaltig produzierter Güter auf Märkten, auf denen diese zunehmend nachgefragt werden. Allerdings kann es Unternehmen unter bestimmten Umständen durch Kooperationen mit Wettbewerbern erleichtert werden, ökologisch nachhaltige Produkte einzuführen. Dies kann z.B. der Fall sein, wenn Informationsasymmetrien hinsichtlich der Produkteigenschaften bestehen. Eine generelle Ausnahme für derartige Nachhaltigkeitsinitiativen empfiehlt die Monopolkommission jedoch nicht. Eine Abwägung von Wettbewerbsschutz und Nachhaltigkeitszielen kann, z.B. auch im Rahmen der Fusionskontrolle, unter Berücksichtigung von externen Effekten, z.B. durch Umweltverschmutzung, erfolgen. Eine explizite Aufnahme von Effizienzabwägungen in der Fusionskontrolle könnte auch in Deutschland kartellrechtlich verankert werden. Bisher ist dies lediglich auf europäischer Ebene der Fall und sorgt dort für eine systematische und einheitliche Prüfung. Die Monopolkommission sieht jedoch zumindest bisher keinen Anlass für eine derartige Gesetzesanpassung.

## Einfluss steigender Energiepreise auf Marktstrukturen

Eine große Rolle in der wirtschaftspolitischen Diskussion zu Nachhaltigkeit und Klimawandel spielen in Deutschland die Energiewende und aktuell steigende Energiepreise. Vor dem Hintergrund einer möglichen Energiekrise im Zuge des russischen Kriegs gegen die Ukraine, aber auch anzunehmender langfristiger Energiekostensteigerungen, stellt sich nicht nur die Frage, wie die Energiewende und die deutschen Klimaziele davon betroffen sein werden, sondern auch, welche Folgen dies für die Marktstrukturen und den Wettbewerb haben wird.[9] Durch steigende Energiepreise steigen speziell die Produktionskosten energieintensiver Industrien in Deutschland, wodurch ein Anstieg der Anbieterkonzentration gerade in diesen Branchen denkbar ist.[10] So sind im April 2022 die Großhandelspreise für Mineralölerzeugnisse im Vergleich zum Vorjahresmonat laut Statistischem Bundesamt um 63 % angestiegen. Bereits im Verlauf der zweiten Ölpreiskrise Ende der 1970er Jahre schieden Anbieter infolge gestiegener Energiepreise und schwacher Konjunktur aus dem Markt aus. 1984 stellte die Monopolkommission fest, dass die Unternehmenskonzentration zwischen 1979 und 1981 in der überwiegenden Zahl der Wirtschaftszweige angestiegen war. Insbesondere auf den Märkten der energieintensiven Wirtschaftszweige ist – ohne staatliche Eingriffe – auch heute eine zunehmende Zahl von Marktaustritten denkbar. Bei einer ausbleibenden Gründungsdynamik, die die Austritte kompensieren könnte, würde dies die Anbieterkonzentration erhöhen. Besondere Aufmerksamkeit ist vor allem dann geboten, wenn die Unternehmenskonzentration in betroffenen Bereichen ohnehin bereits hoch ist, wie z.B. in der Kokerei und Mineralölverarbeitung.

Wie sich Konzentration und Marktmacht in energieintensiven Industrien entwickelt hat, wird aus Abbildung 4

9 Der Begriff der energieintensiven Industrien umfasst im Folgenden all jene Industrien, die Teil des europäischen Emissionshandels sind.

10 Aber nicht nur die einhergehende Teuerung der Produktion, sondern auch die Störung von Lieferketten durch Wirtschaftssanktionen gegenüber Russland sowie Beeinträchtigungen der wirtschaftlichen Aktivität der Ukraine haben das Potenzial, sich negativ auf den Wettbewerb einzelner Märkte auszuwirken.

Abbildung 4

**Energieintensive Industrien mit sinkender Konzentration aber steigenden Preisaufschlägen**

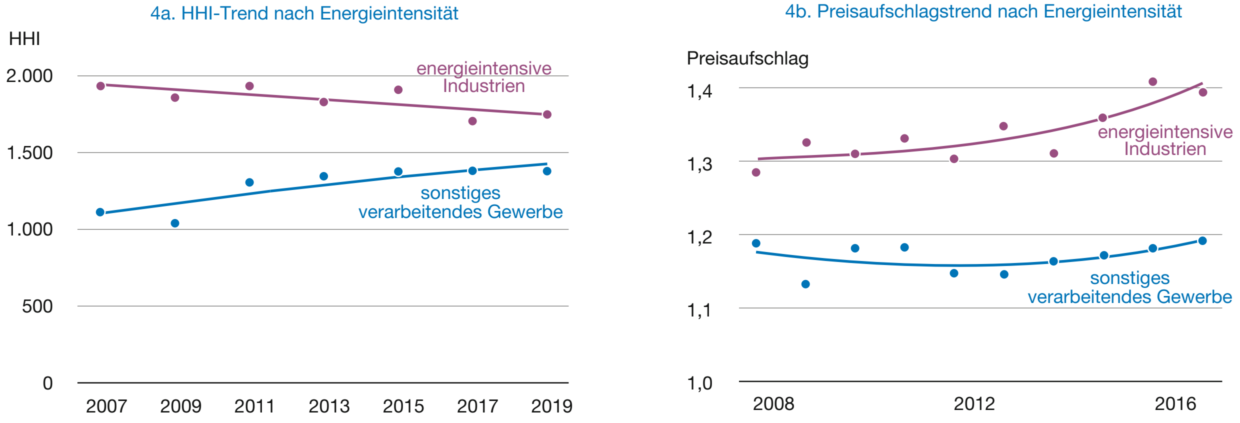

Trends und jährliche Mittelwerte in a) basieren auf HHI für 4-Steller-Wirtschaftszweige nach WZ 2008 und sind umsatzgewichtet. Trends entsprechen REML-Schätzungen von GAM-Modellen mit kubischen Splines.

Quelle: eigene Darstellung nach Monopolkommission (2022), basierend auf einer konzentrationsstatistischen Sonderaufbereitung des Statistischen Bundesamtes auf Basis des Unternehmensregisters sowie der AFiD Panel Industrieunternehmen 2008-2017 und Strukturerhebung im Dienstleistungsbereich 2008-2017.

ersichtlich. Teilabbildung 4a zeigt für den Zeitraum 2007 bis 2019 den HHI energieintensiver Industrien und verdeutlicht, dass die durchschnittliche Unternehmenskonzentration in energieintensiven Industrien zwischen 2007 und 2019 insgesamt um 12 % gesunken ist, während sie im sonstigen verarbeitenden Gewerbe um gut 25 % angestiegen ist.

Neben der Unternehmenskonzentration komplementieren Preisaufschläge das Bild der Wettbewerbslage. Im Kontext steigender Energiepreise ist zu beachten, dass konzentrierte Märkte, die zusätzlich durch hohe Preisaufschläge charakterisiert sind, besser in der Lage sein können, steigende Produktionskosten zu kompensieren. Für die USA wird bereits diskutiert, ob marktübergreifend hohen Unternehmensgewinne für eine Verzögerung eines konjunkturellen Abschwungs verantwortlich sind (Economist, 2022). Marktstrukturelle Effekte infolge steigender Energiepreise sind daher speziell in Bereichen mit geringen Preisaufschlägen zu erwarten. Vor diesem Hintergrund zeigt Abbildung 4b, wie sich die Preisaufschläge energieintensiver Industrien und des sonstigen verarbeitenden Gewerbes in Deutschland zwischen 2008 und 2017 entwickelt haben. Es zeigt sich, dass die Preisaufschläge energieintensiver Wirtschaftszweige des verarbeitenden Gewerbes deutlich höher als im sonstigen verarbeitenden Gewerbe ausfallen und der Anstieg der Preisaufschläge ab 2014 erheblich stärker ausfällt.[11]

11 Für eine abschließende Beurteilung des Preissetzungsspielraums sind zusätzlich insbesondere die Fixkosten in diesen Wirtschaftsbereichen zu berücksichtigen.

Kouvavas et al. (2021) folgen einer ähnlichen Argumentation in ihrer Erklärung der stabil niedrigen Inflation während der Niedrigzinspolitik der vergangenen zwei Jahrzehnte. Sie argumentieren, dass Unternehmen mit hohen Gewinnspannen ihre Preise nach expansiven Schocks weniger stark anheben und hohe Gewinnspannen die beobachtete niedrige Inflation miterklären. Für einen gewissen Kompensationsspielraum könnte in manchen Fällen auch die – trotz Pandemie und russischen Kriegs gegen die Ukraine – zuletzt positive Gewinnentwicklung deutscher Großunternehmen sprechen.[12]

## Fazit

Die beschriebenen Transformationsprozesse haben bereits vielfältige Änderungen der wettbewerbspolitischen Rahmenbedingungen ausgelöst. Dazu zählen Anpassungen des deutschen und europäischen Wettbewerbsrechts im Rahmen der 10. Novelle des Gesetzes gegen Wettbewerbsbeschränkungen (GWB-Digitalisierungsgesetz) und der Digital Markets Act (DMA). Beide stellen gesetzgeberische Reaktionen auf die gewachsene Bedeu-

12 Vgl. FAZ vom 13. Juli 2022, Die Gewinne steigen trotz Krieg und Krise. Ein Blick auf die energieintensiven Unternehmen im Dax 40 – BASF, Bayer, Covestro, HeidelbergCement und Linde – zeigt, dass diese Unternehmen auch im ersten Quartal 2022 trotz der sich zuspitzenden Situation an den Energiemärkten ihre Gewinne steigern konnten. HeidelbergCement verzeichnete in seinem „operating EBIT" im Vergleich zum Vorjahr zwar einen Rückgang um knapp 59 %, im Vorgleich zum ersten Quartal im Prä-Pandemie-Jahr 2019 aber trotzdem noch ein Plus von fast 63 %. Die Entwicklungen für HeidelbergCement könnte darauf zurückzuführen sein, dass HeidelbergCement 2021 seine Mehrheitsbeteiligung von 51 % am kuwaitischen Zement- und Transportbetonunternehmen Hilal Cement verkauft hat, wodurch das „operating EBIT" 2021 ungewöhnlich hoch ausfiel.

tung von Plattformmärkten im Zuge der Digitalisierung dar. Die Suche nach Lösungsmöglichkeiten zur Beherrschung von Plattformmärkten und der Digitalökonomie ist damit zwar noch nicht abgeschlossen, aber wie seitdem aufgenommene Verfahren des Kartellamtes gegen die GAFA-Unternehmen (Google, Amazon, Facebook (Meta) und Apple) zeigen, war dies ein relevanter Schritt zur Modernisierung des deutschen Wettbewerbsrechts im digitalen Zeitalter (Bundeskartellamt, 2021). Aktuell wird zudem eine weitere Verschärfung des deutschen Kartellrechts im Rahmen einer 11. GWB-Novelle angestrebt, bei der auch die Bedeutung von marktstrukturellen Wettbewerbsfaktoren deutlich betont wird (BMWK, 2022).

Neben derartigen Anpassungen des Rechtsrahmens sind vor dem Hintergrund der COVID-19-Pandemie bereits viele staatliche Eingriffe in das deutsche Wirtschaftssystem erfolgt und standen jüngst angesichts hoher Energiepreise unter anderem in Form einer Abschöpfung sogenannter Übergewinne im Mittelpunkt der wettbewerbspolitischen Diskussion. Angesichts der geschilderten Transformationsprozesse werden staatliche Eingriffe und die Forderungen nach diesen in Zukunft vermutlich zunehmen. Dabei gilt es, das Wettbewerbsgeschehen auf den jeweiligen Märkten genau im Blick zu behalten, um die wohl effektivste Antriebskraft zur Transformation der deutschen Wirtschaft, einen funktionierenden Wettbewerb, nutzen zu können. Denn ohne einen funktionierenden, obgleich kanalisierten, Wettbewerb, wird es vermutlich keine Transformation geben, die sowohl in angemessener Geschwindigkeit erfolgt als auch nachhaltig erfolgreich ist. Eine kontinuierliche und sektorübergreifende Beobachtung des Wettbewerbs gewinnt vor diesem Hintergrund an Bedeutung.
